# Round the Hospitals

**Published:** 1920-12-04

**Authors:** 


### ROUND THE HOSPITALS.

The fourth celebration of Birthday Week at the Imperial Nurses' Club, Ebury Street, is a particularly gay one. The programme includes entertainments of some kind every day—in the mornings there is coffee, and music from 10 to 12 for those on night-duty, there are concerts and tea-parties in the afternoon, an annual meeting on Wednesday, December 1, and on Saturday, December 4, progressive games and competitions from 3 to 5.30. On Sunday, December 5, Miss Sybil Thesiger will speak on "The Adventure of the Future." The first day of the week, Monday last, was reserved for an At Home for original members—who joined before 1917—and friends of the Club. The hostesses were Lady Lyttelton (Vice-President), Mrs. Weir (Chairman), and Miss Mayers (Honorary Secretary), and amongst the guests were Dame Sarah Swift, Miss Finch (University College Hospital), Miss Hogg (Guy's Hospital), Miss Amy Hughes, Miss Richardson (Nurses' Missionary League), Miss Rae, and Miss Willcox (King's College Hospital). After tea had been served in the dining-room, the Archdeacon of London dedicated the new lounge which opens out of the hall. In his dedicatory prayer he spoke of the Club and prayed that all who came to rest within this room might, though weary in work, never be weary of work, and that it might be a meeting-place for new friends. The Countess of Selborne then announced the Lounge open, and it was inspected and much admired by the visitors. The colour-scheme of blue and grey is very restful, and the room is pleasantly and comfortably furnished.

Many matrons are assisting the Registrar of the College of Nursing by very kindly forwarding new addresses of their recent staffs when they are known. This help is very much appreciated, and has been the means by which the College has been put into touch with members who have left military and other hospitals, and forgotten to notify their change of address to headquarters. There are still many addresses wanted, and the College will be grateful if other matrons can assist in this way.

The Countess of Selborne took the chair at the annual meeting of the Hampshire County Nursing Association, which was well attended. The County Superintendent, Miss Bröchner, has a good year to her credit. The tide is perceptibly turning and a larger number of candidates for training presented themselves than since war began, but the supply is still inadequate to meet the demands of the local Associations. The grant from the Ministry of Health of £637 is used entirely for training purposes. The work of the Hampshire Voluntary Society for the Care of the Mentally Defective has now been severed from that of the Nursing Association, but the two societies share a motor-car and certain office expenses. A donation of £3,120 from the Red Cross enables the Association to end their year with a balance of over £2,000, which is to be invested until it can be used for establishing a central training-home. Miss Glass, Inspector of Q.V.J.I.N., gave an interesting address, in which she stated that the Jubilee Institute had recommended at a recent conference that the salary should be £63, all found, and calculated this would mean a total of £180 to £200. This, of course, is for fully-trained and certificated nurses.

Mrs. Crewe, who for thirty-nine years was matron of the Royal Lancaster Infirmary, and who retired in July, has just received from grateful patients and friends in Lancaster a sum of £373 15s. 8d., subscribed through a sub-committee of the infirmary, in appreciation of her devoted service and kindness during her long *régime*. The committee of the institution expressed their appreciation of Mrs. Crewe's services in a tangible way when she retired, and this further expression of regard has given great satisfaction to Mrs. Crewe, especially as the handsome sum has been subscribed by all classes, poor and rich alike, in a quiet and unostentatious way.

Sir Napier Burnett, in a timely article which appears in a Sunday paper, brings before his readers the importance of detecting early cases of tuberculosis, and lays great stress on the need for more nurses. He estimates that 90 per cent. of the working classes are cared for in sickness in their own homes, and must depend on the services of district nurses. We believe that far more should be done to make known the need for nursing candidates, and that the Sunday papers offer a valuable means of bringing facts before the women most likely to respond to the call. What is wanted is a publicity department run by some central authority. For hospitals to compete with each other for candidates is a suicidal policy.

Several interesting appointments are recorded under Matrons' and Sisters' Appointments on page viii. of this issue. Amongst them is that of Miss M. K. Coggins to be Matron of the West London Hospital, Hammersmith, succeeding Miss F. E. Nevile, after the latter's service as matron there for upwards of fourteen years. Miss Coggins, who was trained at St. George's Hospital, has since seen much service in the North of England, and her return to the Metropolis will doubtless prove as pleasant to her as it should be of benefit to the hospital, whose nursing department must needs expand with the extensions which are contemplated, which will bring the number of beds to 400. Another interesting appointment is that of sister-tutor, combined with the post of theatre sister, at Beckett Hospital, Barnsley. We are glad to see evidence of a spirit of progress in the nursing department of this institution, and congratulate the authorities on the appointment of Miss M. P. Browne to this office. Her training and experience at the Sheffield Royal Hospital should make her a valuable addition to the staff.

**ROUND THE HOSPITALS**—(*continued*).

The Sanatorium Benefit Sub-Committee of the County of London Insurance Committee have made an important recommendation with reference to the Public Health [Tuberculosis] Bill, which has been introduced into Parliament by the Ministry of Health. The Sub-Committee is of opinion that provision should be made in the Bill for the nursing of tuberculous patients to be undertaken by district or other nursing associations as a part of the treatment of tuberculosis. This would, of course, entail either the employment of special nurses for this particular purpose, or, better still, the grafting of this branch of nursing on to the general work of the nursing associations. More nurses would be required in either case, but in the latter the danger of overlapping and of wasting the nurses' time would be obviated.

The second annual reunion of Westminster Hospital has been arranged for Tuesday, December 7. All former members of the nursing staff are warmly invited, and will be heartily welcomed by the Matron and the present staff between the hours of 4 and 6.30 p.m.

A Conference has been held under Princess Louise at Kensington Palace to arrange for a Ladies' Committee to carry out celebrations in connection with an Empire tribute to the Prince of Wales on December 7, to commemorate his historic tour. Viscount Gladstone, Chairman of the Prince of Wales's Hospital, and also of the Tribute Committee, described the needs of the hospital, for the aid of which the tribute is designed. There is to be a gala night at all places of entertainment on December 7, when during a fifteen-minute interval appropriate songs will be sung and a collection made. Red Cross nurses are being invited to assist.

The Alexandra Hospital for Children with Hip Disease accomplished its removal on November 25 from Queen Square, Bloomsbury, to Swanley, Kent, with great cheerfulness and comfort. The picture Press has some charming scenes illustrating the carriage of the patients, all of whom, to judge from the expression on their faces, highly enjoyed the expedition. We should like to clear all the sick children out of London, to recover tone and health in pure air, leaving only a few cots for accidents, etc., in the general hospitals. The Alexandra Hospital has a new career of usefulness before it in its well-planned country quarters, and we trust the pleasant publicity attaching to the move may have brought its claims to the notice of many desirous of helping forward the good work.

Readers who are interested in the work carried on by the excellent Royal Society for the Protection of Birds, 23 Queen Anne's Gate, S.W. 1, may like to know that some charming Christmas cards are to be had there at very moderate prices, reproducing in colour "The Sea-Blue Bird," by Mr. Roland Green, with or without calendar, and other beautiful studies in bird life. They can be had as postcards also at 1s. 2d. a dozen.

Miss Macdonald, matron-in-chief of the Canadian Army Nursing Service, has addressed a rousing letter in the *Canadian Nurse* to members of her service, including reserve and retired, calling on them, with the end of demobilisation in sight, not to lose sight of each other. The formation of a Canadian Army Nurses' Club is in the air, and meantime Miss Macdonald urges her sisters to furnish some of their interesting and humorous experiences during the war to a separate department in the above journal reserved for the purpose. The appeal, "Come, sisters, I know you are teeming with material," must surely prove irresistible.

An impression which prevails among the public that there are still many unidentified soldiers undergoing treatment in military mental hospitals has met with official contradiction. It is definitely stated that there is no single case in any hospital under military control or any other institution whose identity has not been established. This is an excellent record and bears striking witness to the wonderful improvement which has taken place of late years in dealing with cases of the kind.

News has reached us that the period of training in the nurse training-school at Boksburg Hospital in the Transvaal has been extended from three to four years. This a very healthy sign of a rising standard of nursing education. Boksburg is the centre of the Witwatersrand coal-mining district, and the hospital is one of the leading training-schools for nurses. The additional year has been found necessary on account of the legislation which prescribes an eight-hour day for the nurses, and it is likely to be introduced into other schools, for the course of study cannot be fitted into three years under the conditions imposed by Government.

The Durham County Superintendent Health Visitor reports that in some of the rural districts there are outlying areas which necessitate very long walks for the health visitors concerned. Bicycles are not much help in summer owing to the hills, and in winter they are practically useless. When the staff was small the health visitors seldom visited these remote areas, as they always had plenty of work in the more thickly populated districts in their charge. Experience shows that it is not advisable to neglect these scattered areas, and she accordingly suggests that the County Council should authorise motor-hire for journeys to remote districts. This recommendation is not likely to help anyone, we fear. The cost of motor-hire puts it out of the question to employ this means of transit, either for health visitors or district nurses. But we watch with interest the rapid development of the "scooter" into a safe and practical machine, well adapted to district work, whether for doctors or nurses. It is not too much to say that in hilly districts such a machine would enable one nurse to do the work of two, and thus pay its own expenses.

